# Whole-genome analysis of rotavirus G1P[8] and other Wa-like strains in Mozambican children: evidence of genetic variations of pre-vaccine G1P[8] strains from Manhiça, Mozambique

**DOI:** 10.1099/mgen.0.001522

**Published:** 2025-10-28

**Authors:** Filomena Manjate, Percina Chirinda, Peter Mwangi, Eva D. João, Milton Mogotsi, Marcelino Garrine, Augusto Messa Jr., Delfino Vubil, Nélio Nobela, Karen Kotloff, James P. Nataro, Tacilta Nhampossa, Sozinho Acácio, Goitom Weldegebriel, Jacqueline E. Tate, Umesh Parashar, Jason M. Mwenda, Pedro L. Alonso, Martin Nyaga, Celso Cunha, Inácio Mandomando

**Affiliations:** 1 Centro de Investigação em Saúde de Manhiça (CISM), Maputo 1929, Mozambique; 2 Global Health and Tropical Medicine (GHTM), Instituto de Higiene e Medicina Tropical (IHMT), Universidade Nova de Lisboa (UNL), 1349-008 Lisbon, Portugal; 3 Next Generation Sequencing Unit, School of Biomedical Sciences and Division of Virology, Faculty of Health Sciences, University of Free State, Bloemfontein, South Africa; 4 Zambeze University, Faculty of Environmental Engineering and Natural Resources, Chimoio, Mozambique; 5 Facultat de Medicina i Ciències de la Salut, Universitat de Barcelona (UB), Barcelona, Spain; 6 ISGlobal, Barcelona 08036, Spain; 7 Center for Vaccine Development (CVD), University of Maryland, School of Medicine, Baltimore, MD 21201, USA; 8 Department of Paediatrics, University of Virginia School of Medicine, Charlottesville, VA 22903, USA; 9 Instituto Nacional de Saúde (INS), Ministério da Saúde, Marracuene 1120, Mozambique; 10 African Rotavirus Surveillance Network, Immunization, Vaccines and Development Program, World Health Organization, Regional Office for Africa, Brazzaville P.O. Box 2465, Republic of Congo; 11 Centers for Disease Control and Prevention (CDC), Atlanta, GA 30333, USA

**Keywords:** lineages, rotavirus in Mozambique, Wa-like constellation, whole-genome sequencing

## Abstract

The Rotarix® (GlaxoSmithKline, Rixenstart, Belgium) vaccine was adopted into the Expanded Program on Immunization in Mozambique in September 2015, and G1P[8] strains were frequent pre- and post-vaccine introduction in some regions of the country. In the current study, G1P[8] and other strains (G12P[6], G12P[8] and G9P[8]) circulating between 2008–2012 and 2015 in children with and without diarrhoea were characterized by next-generation sequencing to understand their genetic composition. The G1P[8] strains were compared with Mozambican pre- and post-vaccine strains retrieved from GenBank. All study strains, either from children with or without diarrhoea, were genetically similar and exhibited a typical Wa-like constellation. The Mozambican pre- and post-vaccine G1 strains clustered in two separate lineages: most pre-vaccine strains in lineage I and all post-vaccine strains in lineage II. Meanwhile, all P[8] were in lineage III, although the study strains were in a different cluster from the G1P[8] post-vaccine strains retrieved in GenBank. Analysis of VP7 epitope regions showed amino acid differences such as S123N and M217T in all study strains, while a change N94S was observed in seven strains and K291R in eight strains. Sequence alignment of G1P[8] study strains using mVISTA confirmed that they may have suffered point mutations in all their 11 segments. Our results suggest that host factors are implicated in the inducement and severity of diarrhoea and these factors need further investigation. The changes in some G1 strains at the epitope region emphasize the need for continued whole-genome analysis, as substitutions in this region are reported to affect protein conformation at given positions and the study results may provide data for future studies aiming to understand rotavirus strain evolution post-vaccine introduction.

**Impact Statement**

The G1P[8] is the most common rotavirus combination worldwide and comprises the Rotarix® vaccine. This has generated a lot of attention to expanded research into the potential of G1P[8] strains to develop into vaccine escape mutants post-vaccine introduction. Mozambique uses the Rotarix vaccine, and whole-genome-based studies on this genotype pre- and post-vaccine introduction have been scarce in Manhiça, Mozambique, hindering the assessment of potential mutations in vaccine epitope regions. Amino acid changes at the epitope regions may affect protein conformation at given positions and enhance the virus evolution. In our analysis, we observed amino acid changes inside the epitope region in the VP7 outer capsid protein and point mutations in all 11 genome segments of G1P[8] strains pre-vaccine introduction. These results provide a long-term understanding of the diversity of rotavirus strains in Mozambique, and we provide data useful for comparative studies in the future, aiming to investigate rotavirus genetic changes based on whole-genome analysis, to successfully understand their evolution post-vaccine introduction.

## Data Summary

The authors confirm that all supporting data have been provided within the manuscript or through the supplementary data files.

## Introduction

Despite the availability and global use of rotavirus vaccines [1], rotavirus group A (RVA) is still the major aetiology associated with acute diarrhoea in children under 5 years [2]. Globally, in 2019, it caused over 1,760,113 [interquartile range (IQR): 1,422,645–2,925,372] hospitalizations [3]. According to estimates using data from the Global Burden of Disease, about 19.1% of global diarrhoeal deaths were associated with rotavirus in 2019 [4], with African countries, Oceania and South Asia bearing the highest death rates, between 1990 and 2019 [4]. Four rotavirus vaccines (Rotarix®, GlaxoSmithKline, Rixenstart, Belgium; RotaTeq®, Merck and Co, USA; ROTAVAC®, Bharat Biotech, Hyderabad, India; and ROTASIIL®, Serum Institute of India, Pune, India), pre-qualified by the World Health Organization, are routinely used worldwide [1], following recommendation for vaccine introduction in countries with high disease burden [5]. Mozambique has adopted the use of Rotarix® into the Expanded Program on Immunization since 2015 [6]. This decision was supported by data from the Global Enteric Multicenter Study (GEMS), which was conducted in four sub-Saharan Africa (Kenya, Mali, Mozambique and The Gambia) and three Asian (Bangladesh, India and Pakistan) countries between 2007 and 2012 [7,8]. The GEMS study showed high attributable fraction of rotavirus in Mozambican infants (aged 0–11 months), contributing 35% of moderate-to-severe diarrhoea (MSD) and 20% of less severe diarrhoea (LSD) [7,9].

The dsRNA genome of RVA consists of 11 segments encoding six structural proteins (VP1–VP4, VP6 and VP7) and five to six non-structural proteins (NSP1–NSP5/NSP6) [10]. Genotype designation is mainly based on the two outer capsid proteins, VP7 (the glycoprotein G) and VP4 (protease-sensitive P), which segregate independently and stimulate the production of neutralizing antibodies during infection [11]. Based on VP7 and VP4 classification, combinations such as G1P[8], G2P[4], G3P[8], G4P[8], G9P[8] and G12P[8] were commonly found circulating and causing infection worldwide [12,14]. Besides this, the genotypes are more completely classified based on all the other gene segments [11,15], and through whole-genome sequencing (WGS), three main constellations have been identified. G1-P[8]-I1-R1-C1-M1-A1-N1-T1-E1-H1 is the prototype Wa-like constellation [11], although other genotypes, such as G3, G4 and G9, in combination with P[8] are also Wa-like, with a porcine origin [11]. The prototype G2-P[4]-I2-R2-C2-M2-A2-N2-T2-E2-H2 designated Ds-1-like constellation, and the prototype G3-P[9]-I3-R3-C3-M3-A3-N3-T3-E3-H3 designated Au-like [16].

Nevertheless, by WGS, many rare combinations have been uncovered, including atypical G1P[8], G3P[8] and G9P[8] bearing Ds-1-like constellation [17,19]. Reassortments are frequently observed in rotavirus, due to its segmented genome, and the lack of proof-reading activity of RNA-dependent RNA polymerase allows the occurrence of high mutation rates [20], which results in the emergence of new rotavirus strains and distinct lineages [21]. Rotarix vaccine, which is composed of a G1P[8] strain, was reported to have lower effectiveness against non-vaccine strains, compared to vaccine strains, in a pooled secondary analysis of clinical trials and post-licence studies [22]. Whether this is associated with vaccine selective pressure or natural genotype fluctuation remains unclear [23].

In Mozambique, surveillance studies have been conducted to provide the burden of rotavirus [6,8, 9, 24], associated genotypes circulating pre- and post-vaccine introduction [25,28], including characterization of genotypes by WGS in humans [29,33] and animals [34,35]. Additionally, the diversity of rotavirus strains in the country through interspecies transmission has been demonstrated based on WGS [33]. Among VP7 genotypes, G1 is reported to be the most predominant strain in humans worldwide, representing 40% of circulating strains pre-vaccine [36], and between 22 and 33% of strains post-vaccine introduction [37,38]. The combination G1P [8] was identified as one of the most frequent circulating in some regions of Mozambique pre- and post-vaccine introduction [26,27]. Nevertheless, the vaccine effectiveness (VE) against G1P[8] infections in Mozambican children is low, about 30% (95% CI, −355, 81) and 35% (95% CI, −35, 67) for non-G1P[8] infections [39]. The impact of reassortment and recombination events on the low VE observed in Mozambican children is unclear [39].

Despite the availability of data describing rotavirus strain in Mozambique, this is limited to a few regions of the country and based on strains causing MSD and strains characterized few years pre-vaccine introduction (2011–2014) [27,29, 31]. Furthermore, WGS data for G1P[8] strains from Manhiça – both pre- and post-vaccine introduction – are lacking, hindering the assessment of potential mutations in vaccine epitope regions. In this study, we characterized rotavirus G1P[8], and other strains (G12P[6], G12P[8] and G9P[8]) detected pre-vaccine introduction (2008–2012 and 2015) in children with diarrhoea (MSD and LSD) and without diarrhoea (healthy children recruited in the community as controls) in the Manhiça District, Mozambique, to explore their evolutionary lineages and genetic composition.

## Methods

### Strains and site description

Sequencing was performed to rotavirus-positive samples detected pre-vaccine introduction (2008–2012 and 2015) in the GEMS study (*n*=23) and in the surveillance of diarrhoeal diseases (*n*=1), conducted by the Centro de Investigação em Saúde de Manhiça, within the demographic and morbidity surveillance platforms implemented since 1996 [40,41]. All the samples had previous results of VP7 (G) and VP4 (P) genotypes characterized by conventional methods [42,46], as demonstrated in Table S1, available in the online Supplementary Material. Both GEMS and the surveillance of diarrhoeal diseases conducted case-control studies and collected stool samples of children under 5 years as previously described [6,9,26, 30]. In brief, children were enrolled as MSD cases if they presented at the healthcare facility with episodes of diarrhoea defined as three or more abnormal loose stools in 24 h, requiring intravenous rehydration and hospitalization, while LSD were diarrhoea cases attended at the outpatient visits, not requiring hospitalization [7,9,26, 30]. The rotavirus-positive samples analysed in the current study were distributed as follows: 9 G1P[8] (8 from MSD and 1 from a child without diarrhoea), 1 G9P[8] from a child with MSD, 2 G12P[6] (1 LSD and 1 from a child without diarrhoea) and 12 G12P[8] (6 MSD, 3 LSD and 3 from children without diarrhoea). Additionally, Mozambican pre- and post-vaccine G1P[8] strains retrieved from GenBank (https://www.ncbi.nlm.nih.gov/genbank/) were included in the dataset for comparisons. A full description of the characteristics of the enrolled children from whom the samples were collected, including sex and age, is presented in Table S1.

### RNA extraction and purification for WGS

The rotavirus dsRNA was extracted using 1 ml faecal suspension using TRIzol™ (Invitrogen, Carlsbad, CA) based on the protocol described by Potgieter *et al*. [47], with slight modifications previously described [30,48]. Lithium chloride (Sigma, St. Louis, MO, USA) was used for RNA precipitation [30,48] to guarantee complete removal of single RNA [49]. All the extracted RNA was purified using the MinElute gel extraction kit (QIAGEN, Hilden, Germany), according to the kit’s protocol, and ~5 µl of the purified RNA of each sample was evaluated for integrity using electrophoresis on a 1% agarose gel (Bioline, UK), stained with ethidium bromide. The bands were visualized on an ultraviolet transilluminator (Bio-Rad Laboratories, Hercules, CA, USA).

### Synthesis of the cDNA and WGS

Approximately 13 µl of the extracted RNA was transcribed to cDNA using the Maxima H Minus Double-Stranded cDNA Synthesis Kit (Thermo Fischer Scientific, Waltham, MA) following the instructions of the manufacturer as described [18,30, 50]. The transcribed cDNA was purified using the MSB Spin PCRapace Purification Kit (Stratec Molecular, Berlin, Germany) as per the manufacturer’s instructions. Afterwards, DNA libraries were prepared using the Nextera XT DNA Library Preparation Kit (Illumina, San Diego, CA, USA) according to the manufacturer’s protocol, followed by paired-end WGS with the MiSeq Reagent Kit v.3 on the Illumina MiSeq platform for 600 cycles (301 bp × two read length) (Illumina, San Diego, CA, USA). All the procedures were conducted at the University of the Free State-Next Generation Sequencing (UFS-NGS) Unit, as previously described [18,30, 50].

### Assembly of the genome

The sequence quality control was conducted for all raw sequencing data as previously described [30]. In summary, the sequences were trimmed for low quality and short reads removal, using Geneious Prime (v.2022.0.1) software. Plots of ‘per base sequence quality plot’ with distribution of Q30 were considered suitable for analysis. Subsequently, *de novo* assembly was performed using the software CLC Bio Genomics Workbench (v.22.0). The resulting contigs were mapped to generate complete consensus sequences using the prototype Wa-like G1P [8] reference strain, RVA/Human-tc/USA/Wa/1974 /G1P [8] (accession nos. JX406747–JX406757) using the CLC Bio Genomics Workbench (v.22.0) software and Geneious Prime (v.2022.0.1) software as a complementary tool, as previously described [30,50].

### Determination of whole-genome constellations

The genotype of each of the 11 genome segments was determined using the online tool, Virus Pathogen Database and Analysis Resource (ViPR) (accessed on 15 December 2022) [51].

### Phylogenetic analysis

ORFs of each of the 11 segments of the study strains and strains retrieved from GenBank were aligned as described earlier [30], using the Multiple Sequence Comparison by Log Expectation (muscle) algorithm in the Molecular Evolutionary Genetic Analysis (mega) version 11 [52]. The optimal evolutionary model for each gene segment was determined based on the Akaike information criterion (corrected), with the following models obtained: Tamura 3-parameter+gamma distributed invariable sites (T92+G+I) for G1 and P[8]; T92+gamma distributed (G) for G9, VP6, NSP1-NSP3 and NSP5/6; T92+invariable sites (I) for G12 and NSP4; the Tamura-Nei+gamma distributed (TN93+G+I) for P[6] and VP2; the general time-reversible+G+I (GTR+G+I) for VP1 and VP3. The trees were generated with maximum likelihood phylogenetic method for each segment, using 1,000 bootstrap replicates for estimating the tree topology (branch support), in mega version 11 [52]. Nodes supported by bootstrap values greater than or equal to 70% were considered consistent and strong for the phylogenetic analysis. Pairwise genetic distance matrices were calculated using the p-distance algorithm of mega version 11 [52]. The DS-1-like G2P [4] strain was used as the outgroup in the phylogenetic analysis. All the trees were visualized and edited using FigTree v.1.4.4.

### VP7 and VP4 epitope analysis

The amino acid residues of VP7 (G1) and VP4 (P[8]) study strains were aligned and mapped against cognate neutralization epitopes of the Rotarix, accession nos. JN849114 (VP7) and JN849113 (VP4) using mega version 11 [52]. VP7 possesses three established neutralization epitopes, 7–1 a, 7-1b and 7–2, comprising a total of 29 aa (14 residues from 7 to 1 a sub-unit, 6 from 7-1b and 9 from 7 to 2) [53], and the VP4-neutralizing epitope regions are composed of 37 aa residues [54]. The VP4 spike protein is cleaved by trypsin to segregate into two distinct proteins, the VP8* and VP5* subunits [55], where VP8* is constituted of four regions (8–1 to 8–4) and VP5* is constituted of five regions (5–1 to 5–5) [54].

### Reassortment analysis

To investigate possible reassortments, the ORFs of the 11 genome segments of each sample (G1P[8]) were concatenated using Geneious Prime (v.2022.0.1) [56], and the concatenated sequences aligned using the online server mVISTA (http://www-gsd.lbl.gov/vista), applying the Shuffle-LAGAN global alignment, which allows the detection of rearrangements and inversions in sequences (http://www-gsd.lbl.gov/vista). A random selection was performed to select one G1P[8] strain detected post-vaccine introduction in Mozambique to serve as a reference strain, through a randomized list in Excel, using the RAND formula.

## Results

### Whole-genome constellation analysis

Analysis of the whole-genome constellation of all study strains (*n*=24) exhibited the typical Wa-like genotype constellation (I1-R1-C1-M1-A1-N1-T1-E1-H1) (Table 1). WGS analysis statistics for the 11 genome segments of G1P[8] and other study strains are summarized in Table S2. The respective ORFs were deposited in GenBank under accession nos. PQ762216–PQ762479.

**Table 1.** Genotype constellations of the 11 segments of G1P[8], G12P[6], G12P[8] and G9P[8] strains in children with and without diarrhoea in Manhiça, Mozambique

Strain	Type of subjects	Year	Period	Genotypes by NGS										
				VP7	VP4	VP6	VP1	VP2	VP3	NSP1	NSP2	NSP3	NSP4	NSP5/NSP6
RVA/Human-wt/MOZ/MAN-300256/2008 /G1P[8]	MSD	2008	Pre-vaccine	G1	P[8]	I1	R1	C1	M1	A1	N1	T1	E1	H1
RVA/Human-wt/MOZ/MAN-300299/2008 /G1P[8]	Children without diarrhoea	2008		G1	P[8]	I1	R1	C1	M1	A1	N1	T1	E1	H1
RVA/Human-wt/MOZ/MAN-300329/2008 /G1P[8]	MSD	2008		G1	P[8]	I1	R1	C1	M1	A1	N1	T1	E1	H1
RVA/Human-wt/MOZ/MAN-300220/2008 /G1P[8]	MSD	2008		G1	P[8]	I1	R1	C1	M1	A1	N1	T1	E1	H1
RVA/Human-wt/MOZ/MAN-300405/2008 /G1P[8]	MSD	2008		G1	P[8]	I1	R1	C1	M1	A1	N1	T1	E1	H1
RVA/Human-wt/MOZ/MAN-302545/2009 /G1P[8]	MSD	2009		G1	P[8]	I1	R1	C1	M1	A1	N1	T1	E1	H1
RVA/Human-wt/MOZ/MAN-302509/2009 /G1P[8]	MSD	2009		G1	P[8]	I1	R1	C1	M1	A1	N1	T1	E1	H1
RVA/Human-wt/MOZ/MAN-310422/2012 /G1P[8]	MSD	2012		G1	P[8]	I1	R1	C1	M1	A1	N1	T1	E1	H1
RVA/Human-wt/MOZ/MAN-310425/2012 /G1P[8]	MSD	2012		G1	P[8]	I1	R1	C1	M1	A1	N1	T1	E1	H1
RVA/Human-wt/MOZ/MAN-310450/2012 /G12P[6]	Children without diarrhoea	2012		G12	P[6]	I1	R1	C1	M1	A1	N1	T1	E1	H1
RVA/Human-wt/MOZ/MAN-320713/2012 /G12P[6]	LSD	2012		G12	P[6]	I1	R1	C1	M1	A1	N1	T1	E1	H1
RVA/Human-wt/MOZ/MAN-302160/2009 /G12P[8]	MSD	2009		G12	P[8]	I1	R1	C1	M1	A1	N1	T1	E1	H1
RVA/Human-wt/MOZ/MAN-302543/2009 /G12P[8]	MSD	2009		G12	P[8]	I1	R1	C1	M1	A1	N1	T1	E1	H1
RVA/Human-wt/MOZ/MAN-302433/2009 /G12P[8]	Children without diarrhoea	2009		G12	P[8]	I1	R1	C1	M1	A1	N1	T1	E1	H1
RVA/Human-wt/MOZ/MAN-302427/2009 /G12P[8]	Children without diarrhoea	2009		G12	P[8]	I1	R1	C1	M1	A1	N1	T1	E1	H1
RVA/Human-wt/MOZ/MAN-302466/2009 /G12P[8]	Children without diarrhoea	2009		G12	P[8]	I1	R1	C1	M1	A1	N1	T1	E1	H1
RVA/Human-wt/MOZ/MAN-303555/2011 /G12P[8]	MSD	2011		G12	P[8]	I1	R1	C1	M1	A1	N1	T1	E1	H1
RVA/Human-wt/MOZ/MAN-303550/2011 /G12P[8]	MSD	2011		G12	P[8]	I1	R1	C1	M1	A1	N1	T1	E1	H1
RVA/Human-wt/MOZ/MAN-303545/2011 /G12P[8]	MSD	2011		G12	P[8]	I1	R1	C1	M1	A1	N1	T1	E1	H1
RVA/Human-wt/MOZ/MAN-320717/2012 /G12P[8]	LSD	2012		G12	P[8]	I1	R1	C1	M1	A1	N1	T1	E1	H1
RVA/Human-wt/MOZ/MAN-320725/2012 /G12P[8]	LSD	2012		G12	P[8]	I1	R1	C1	M1	A1	N1	T1	E1	H1
RVA/Human-wt/MOZ/MAN-320790/2012 /G12P[8]	LSD	2012		G12	P[8]	I1	R1	C1	M1	A1	N1	T1	E1	H1
RVA/Human-wt/MOZ/MAN-310426/2012 /G12P[8]	MSD	2012		G12	P[8]	I1	R1	C1	M1	A1	N1	T1	E1	H1
RVA/Human-wt/MOZ/MAN-1552518.5/2015 /G9P[8]	MSD	2015		G9	P[8]	I1	R1	C1	M1	A1	N1	T1	E1	H1

Green colour indicates Wa-like genotype constellation.

### Sequence and phylogenetic analysis of the VP7 (G1) genome segments

**Fig. 1.**
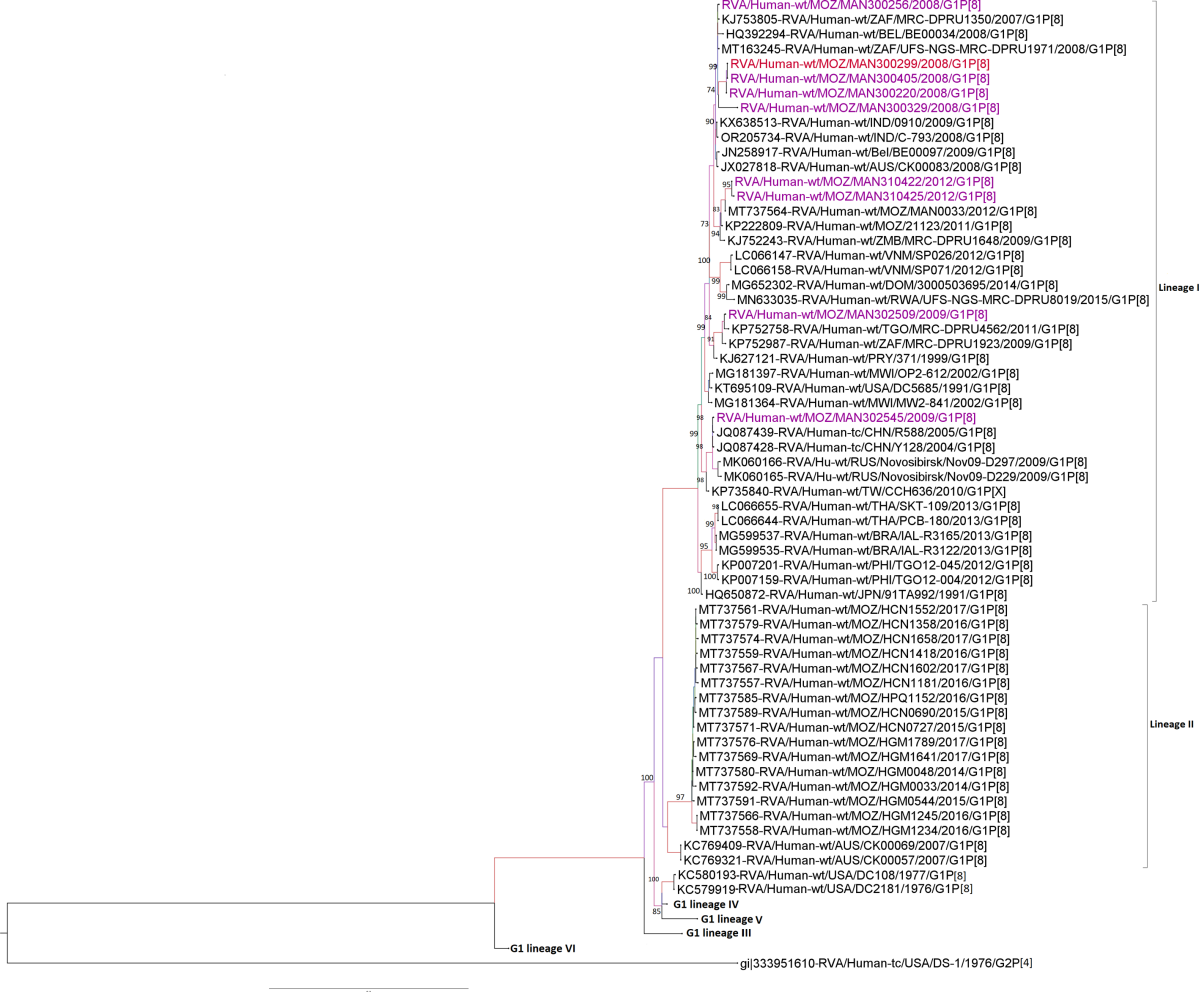
Maximum likelihood phylogenetic tree based on the open reading frames (ORFs) of the VP7 (G1) study strains, with representatives of previously described lineages (I–VI) [67]. Bootstrap values ≥70% are shown adjacent to the nodes. Purple coloured taxa indicate MSD study strains and a red strain of a child without diarrhoea. The DS-1-like RVA strain from the USA is the outgroup.

All the pre-vaccine strains studied clustered in lineage I, composed mainly of sequences from Africa, Europe, Asia and Latin America. Two MSDs (300220 and 300405) and one strain from a child without diarrhoea (300299) from 2008 clustered together, sharing 99.9–100.0% of nt and 100.0% aa similarities. Two other strains from MSD children (310422 and 310425) from 2012 were closely related, sharing 99.8% nt and 99.7% aa similarities (Fig. 1 and Table S3). These two strains were in the same cluster with other Mozambican pre-vaccine strains (MOZ/21123/2011 /G1P[8] and MOZ/MAN0033/2012), with identities ranging from 98.7–99.4% nt and 98.7–99.0% aa (Fig. 1 and Table S3). Meanwhile, the other Mozambican G1 strains circulating between 2014 and 2017 (pre- and post-vaccine introduction) retrieved from the GenBank were in lineage II and had similarities ranging between 91.2–92.9% nt and 93.0–94.6% aa with the study strains (Fig. 1 and Table S3).

### VP7 (G1) Neutralization epitopes’ analysis

**Fig. 2.**
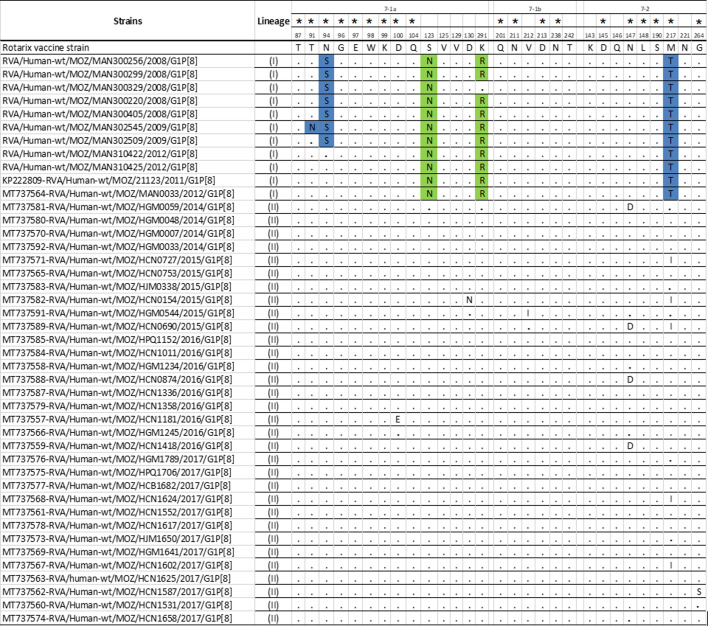
Alignment of antigenic residues in VP7 (G1) [53] of the study strains (from pre-vaccine), with Rotarix® and G1 pre-and post-vaccine strains from Mozambique retrieved in the GenBank. Amino acids, which are different in the study strains with Rotarix®, are highlighted in green and blue colours. A star (*) in the amino acid positions indicates those amino acid changes that were reported to escape neutralization with monoclonal antibodies [53]. Blue indicates amino acid changes in the position demonstrated to escape neutralization with monoclonal antibodies [53]. Conserved residues among the studied strains, pre- and post-vaccine strains from Mozambique and Rotarix® are indicated with a black dot (.).

The nine study strains and the other two Mozambican strains (MOZ/MAN0033/2012 /G1P[8] and MOZ/21123/2011 /G1P[8]) that clustered in lineage I in the phylogenetic analysis, and other G1 Mozambican pre- and post-vaccine strains in lineage II, were aligned and mapped against cognate neutralization epitopes of the Rotarix^®^ strain. Two amino acid differences (S123N and M217T) were identified in all nine study strains, including the two closest pre-vaccine G1 strains retrieved from GenBank. One amino acid difference (N94S) was observed among 7/9 study strains. Additionally, one difference (K291R) was also observed in 8/9 study strains, including the two closest pre-vaccine strains retrieved from GenBank. A single amino acid change was also observed in one study MSD strain, namely T91N (302545) (Fig. 2). Various amino acid residues were similar in the VP7 epitopes of the study strains and the corresponding epitope of the other pre- and post-vaccine G1 strains from Mozambique retrieved from GenBank (Fig. 2).

### Sequence and phylogenetic analysis of the VP7 (G9 and G12) genome segments

**Fig. 3.**
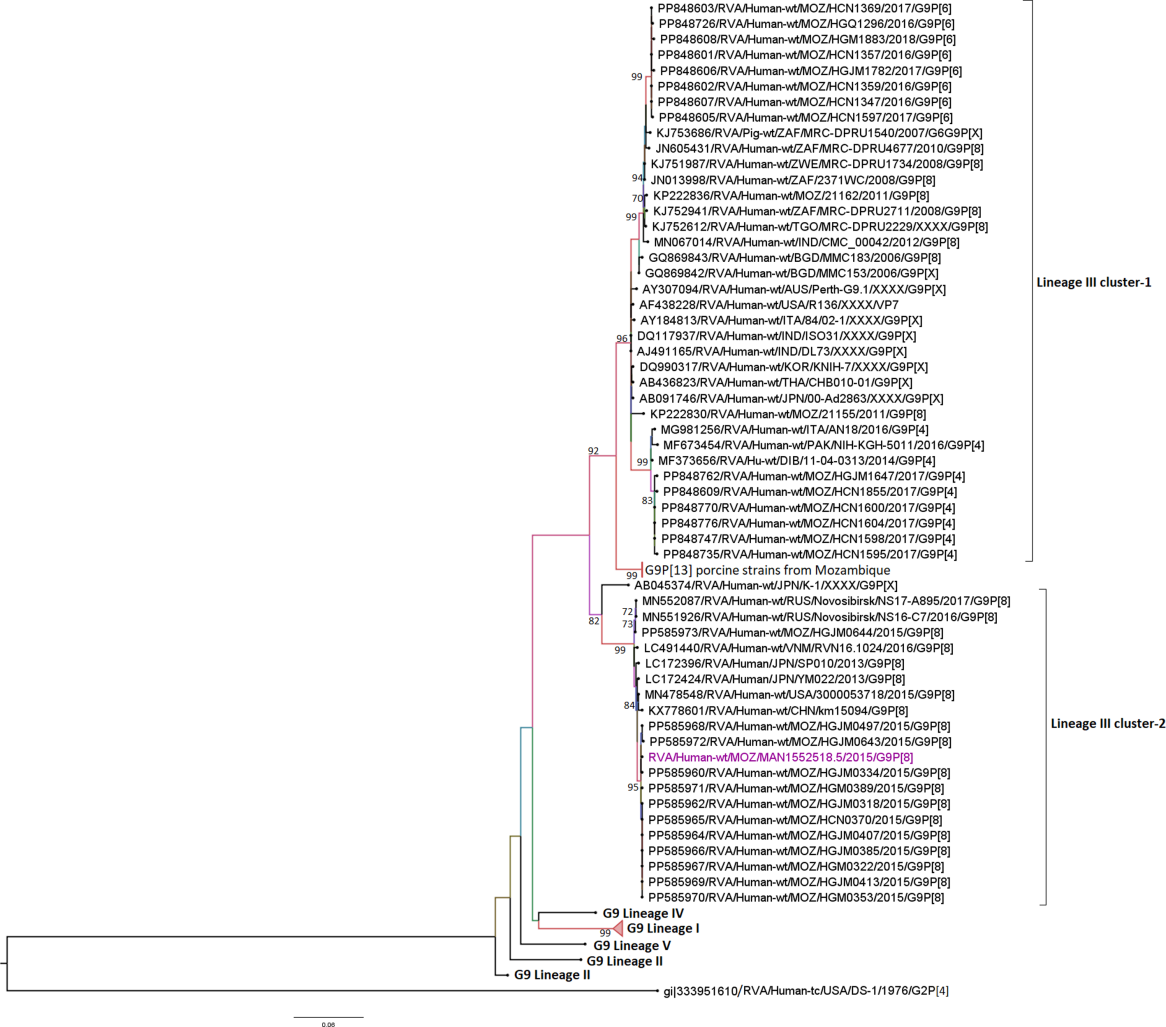
Maximum likelihood phylogenetic tree based on the ORFs of the VP7 (G9) study strain, based on previously described lineages [68,69]. Bootstrap values ≥70% are shown adjacent to the nodes. Purple-coloured taxa indicate the MSD studied strain. The DS-1-like RVA strain from the USA is the outgroup.

The only G9 strain studied was detected in a child with MSD (MAN1552518.5), and it clustered into lineage III, cluster 2. This lineage comprises strains predominantly circulating in African and Asian countries and a few other regions, including the USA and Russia. The sole studied strain was close to strains circulating in Mozambique in 2015, with similarities ranging from 99.2 to 99.8% of nt and 98.8 to 99.7% of aa. This strain was in a different cluster from G9 strains circulating in Mozambique between 2016 and 2018, sharing identities ranging from 91.2 to 92.2% of nt and 94.5 to 94.8% aa compositions (Fig. 3 and Table S4).

**Fig. 4.**
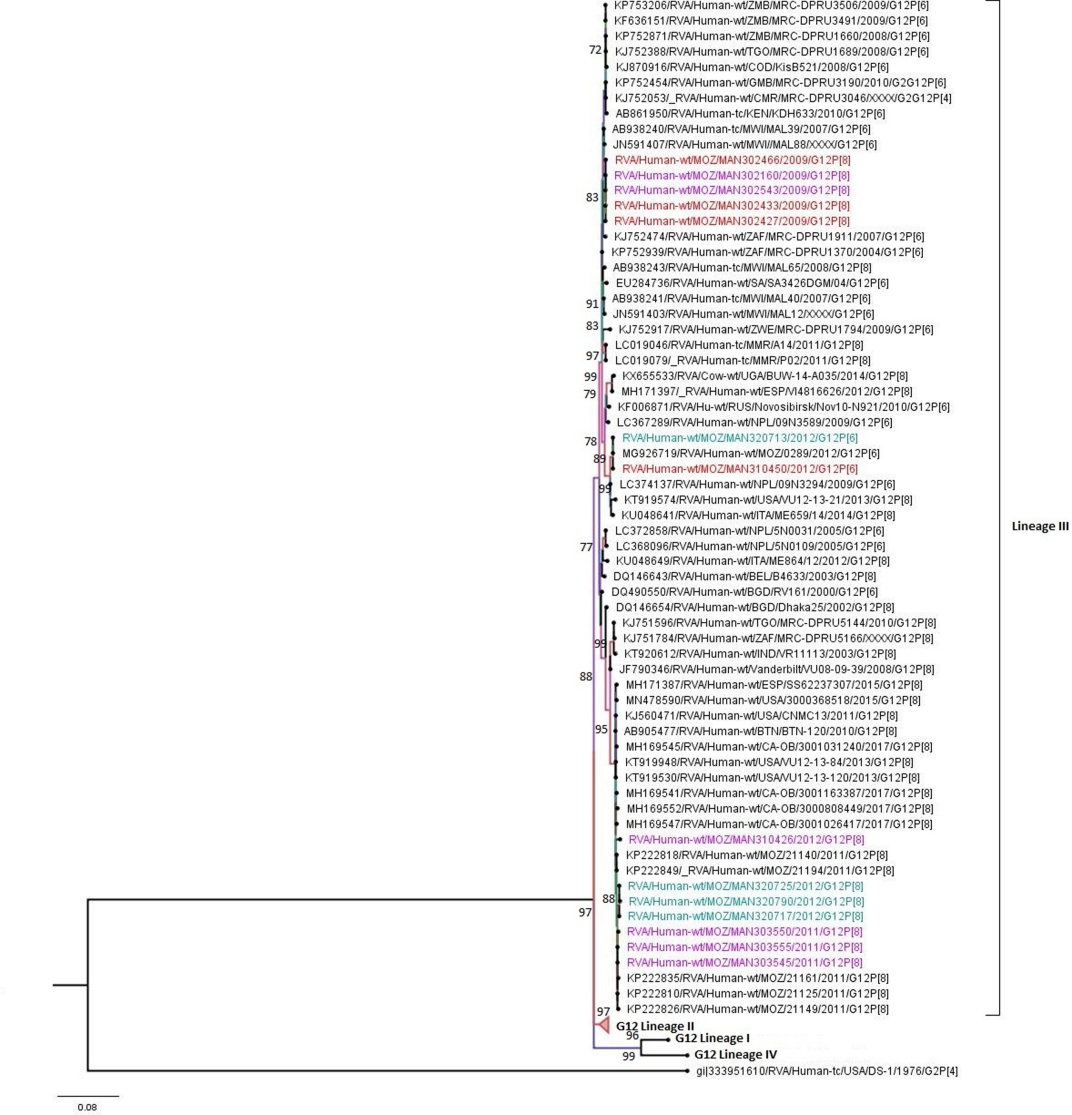
Maximum likelihood phylogenetic tree based on the ORFs of the VP7 (G12) study strains, based on previously described lineages [70]. Bootstrap values ≥70% are shown adjacent to the nodes. Purple coloured taxa indicate MSD study strains, green LSD study strains and red strains of children without diarrhoea. The DS-1-like RVA strain from the USA is the outgroup.

Conversely, all 14 G12 study strains clustered into lineage III and formed two major clusters. The first comprised five strains, two MSD (302160 and 302543) and three from children without diarrhoea (302466, 302433 and 302427), with overall identities ranging from 99–100.0% nt and 99.7–100.0% aa. Moreover, the study strains in this cluster were close to strains that circulated in Malawi, Kenya and South Africa between 2004 and 2010, with identities ranging from 99.2 to 99.8% nt and 99.0 to 100.0% aa. The second cluster comprised six strains, three MSD (303550, 303555 and 303545) and three LSD (320725, 320790 and 320717) sharing 99.5–100.0% nt and 99.0–100.0% aa identities among them. This cluster also comprised G12 strains circulating in Mozambique in 2011, sharing 99.5–100.0% nt and 99.0–100.0% aa similarities with the study strains (Fig. 4 and Table S5).

### Sequence and phylogenetic analysis of the VP4 (P[6] and P[8]) genome segment

**Fig. 5.**
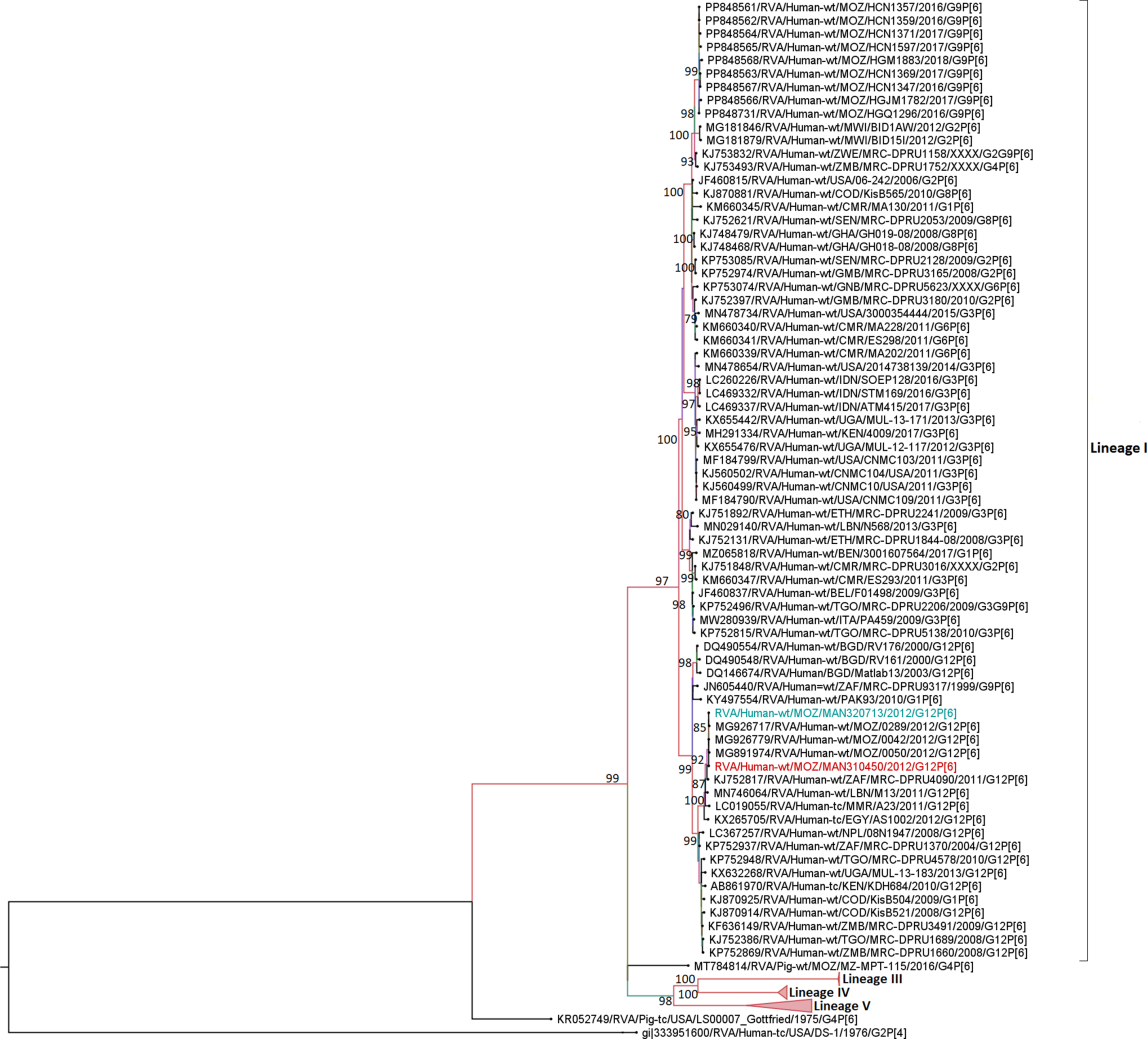
Maximum likelihood phylogenetic tree of the ORFs of the VP4 (P[6]) study strains, constructed based on the previously established five lineages (I–V) [70]. Bootstrap values ≥70% are shown adjacent to the nodes. Purple coloured taxa indicate the MSD studied strain and green LSD study strain. The DS-1-like RVA strain from the USA is the outgroup.

The two studied P[6] strains, one LSD (320713) and one strain from a child without diarrhoea (310450), clustered in lineage I, which comprises most of the P[6] strains circulating worldwide. The strains studied were close to strains that circulated in Mozambique in 2012 (MOZ/0042/2012 /G12P[6], MOZ/0050/2012 /G12P[6] and MOZ/0289/2012 /G12P[6]) sharing 99.9% of nt and aa ranging from 99.6 to 99.9% identities among them. Furthermore, the same cluster also contained strains circulating in Lebanon, Myanmar and Egypt from 2011 to 2012, sharing identities ranging from 99.0 to 99.7% nt and 97.1 to 99.6% aa with the study strains. Meanwhile, the study strains were distant from P[6] detected post-vaccination in Mozambique (2016–2018), with identities ranging from 95.4 to 95.65% of nt and 87.3 to 88.0% of aa (Fig. 5 and Table S6).

**Fig. 6.**
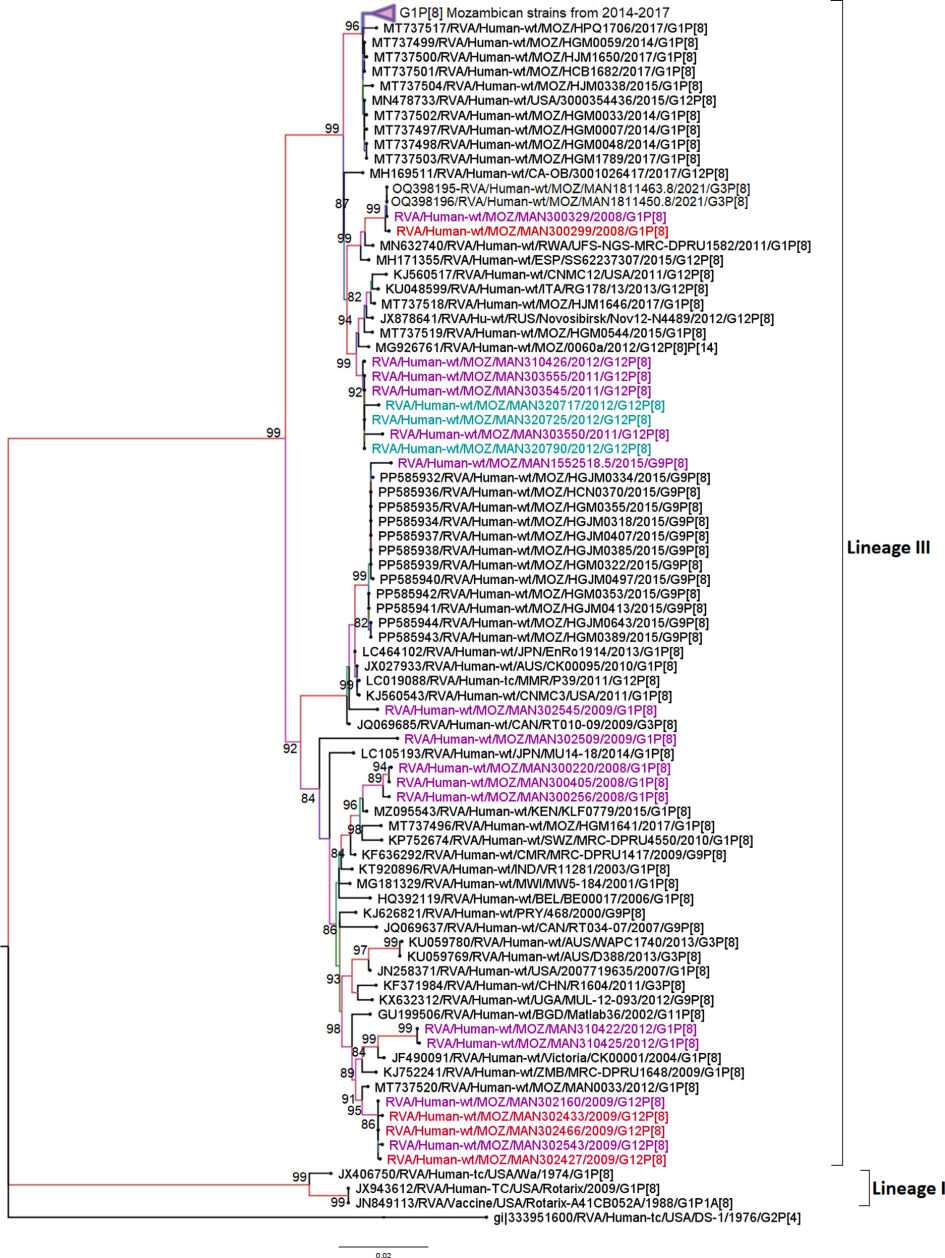
Maximum likelihood phylogenetic tree based on the ORFs of VP4 (P[8]) study strains, constructed based on previously established lineages [71]. Bootstrap values ≥70% are shown adjacent to the nodes. Purple coloured taxa indicate MSD study strains, green LSD study strains and red strains of children without diarrhoea. The DS-1-like RVA strain from the USA was included as an outgroup.

The 22 P[8] study strains (15 MSD, 3 LSD and 4 strains from children without diarrhoea) fell into lineage III, where they formed sub-clusters, either of MSD and LSD strains, MSD and strains from children without diarrhoea or MSD alone, with identities ranging from 99.9 to 100.0% nt and 99.8 to 100.0% among them. In some of the clusters, the strains were close to strains circulating in Manhiça in 2021 (MOZ/MAN1811450.8/2021/G3P[8] and MOZ/MAN1811463.8/2021/G3P[8]) presenting 99.9% nt and 99.8% aa identities, while in other clusters, they were close to other strains circulating in Mozambique from 2012, 2015 and one from 2017, Russia, Rwanda, Italy and Spain, sharing identities ranging from 99.0 to 99.6% nt and 97.0 to 99.0% aa. Conversely, other P[8] strains circulating in Mozambique from 2014 to 2017 were in a distant cluster from all the study strains, with identities ranging from 95.1 to 98.7% nt and 89.1 to 98.1% aa (Fig. 6 and Table S7).

### Analysis of the VP4 neutralization epitopes

**Fig. 7.**
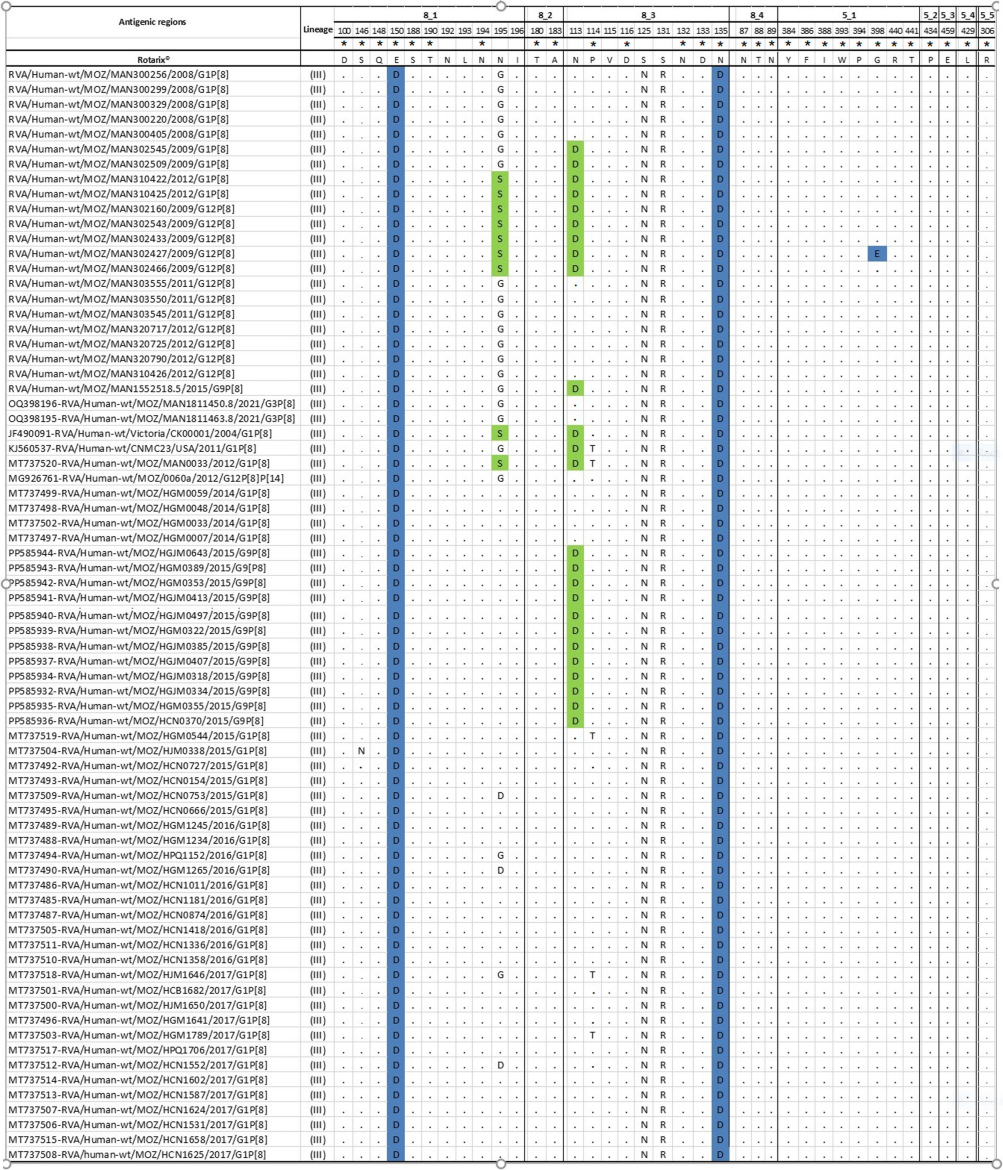
Alignment of the amino acid based on the antigenic epitopes (8–1, 8–2, 8–3, 8–4-VP8*subunit and 5–1, 5–2, 5–3, 5–4, 5–5-VP5* subunit) of VP4 [54,55] of 22 P[8] study strains, compared to P[8] vaccine components of Rotarix®. Amino acids highlighted in light green represent differences when comparing the study strains and other strains to the vaccine strain. Amino acid changes that have been shown to escape neutralization with monoclonal antibodies are indicated with a star (*) [54,55]. Blue colour indicates amino acid changes in the position reported to escape neutralization with monoclonal antibodies [54,55]. Conserved residues among the study strains, pre- and post-vaccine strains from Mozambique and Rotarix are indicated with a black dot (.).

The 22 study strains and additional strains in lineage III that clustered with them in the P[8] phylogenetic tree, including P[8] Mozambican pre- and post-vaccine strains, were aligned and mapped against the neutralization epitopes of the Rotarix^®^ strain. Four amino acid changes (E150D, S125N, S131R and N135D) were observed between the 22 study strains and the strains included in the analysis compared to the Rotarix®. Additionally, changes were observed in residue 195, with a change of N195S distinct between the study strains and the Rotarix® strain, and a change from asparagine (N) to glycine (G) observed in the study strains and other strains retrieved from GenBank. At the same time, a change N113D was observed in 10/22 of the study strains. There was an exclusive change G398E in one of the study strains compared to the Rotarix strain (Fig. 7).

### Sequence analysis and phylogenetic analysis of the VP1–VP3, VP6 and NSP1–NSP5/6 genomes

The phylogenetic evolutionary relationship of VP1–VP3, VP6 and NSP1–NSP5/6 genome segments was performed for all the study strains (*n*=24). In all the genome segments, the strains displayed the same topology, in which three major clusters were formed. One was composed of the cognate G12P[8] strains from MSD and from children without diarrhoea, circulating in 2009. Another included cognate G1P[8] strains from MSD and one strain from a child without diarrhoea circulating in 2008 and, lastly, one of the cognate G12P[8] strains from MSD and LSD circulating in 2012. Additionally, in all the trees, the study strains were close to strains circulating in Africa (Malawi, South Africa, Kenya, Zimbabwe, Uganda, Togo, Congo and Cameroon) sharing identities ranging from 96.7 to 100% for both nt and aa and Asia (India, China and Japan) with identities ranging from 92.1 to 100% nt and 96.3 to 100% aa. They were also close to strains from some European countries (Hungary, Russia and Belgium) sharing identities ranging from 99.1 to 99.9% nt and 98.2 to 99.8% aa and from the USA and Brazil sharing 100% similarity at both nt and aa (Figs S1–9 and Tables S8–16).

Moreover, in all trees, the study strains were close to four Mozambican strains (MOZ/HGM0033/2014/G1P[8], MOZ/0289/2012/G12P[6], MOZ/0042/2012/G12P[6] and MOZ/0060 a/2012/G12P[8]P[14]) sharing identities ranging from 99.0 to 100% nt and 99.1 to 100% aa. One strain MAN1552518.5/2015/G9P[8] was closely related to strains circulating in the country in the same period, sharing identities ranging from 98.1 to 100% nt and 97.7 to 100% aa, while two study strains (MAN302545/G1P[8] and MAN302509/G1P[8]) were not closely related to the other study strains in all the genome segments (except in VP[6] for MAN302545). Nevertheless, in all trees, except in NSP2 and NSP5/6, the study strains clustered separately from two post-vaccine strains previously characterized in the study site in 2021 (MOZ/MAN1811463.8/2021/G3P[8] and MOZ/MAN1811450.8/2021/G3P[8]), sharing with the study strains identities between 87.2–98.3% nt and 88.0–97.9% aa. Besides them, other Mozambican strains circulating between 2014 and 2017 were also in a different cluster, sharing identities ranging from 88.0 to 99.0% nt and 81.5 to 99.0% aa with the study strains (Figs S1–9 and Tables S8–16).

### Reassortment analysis of G1P[8] strains

**Fig. 8.**
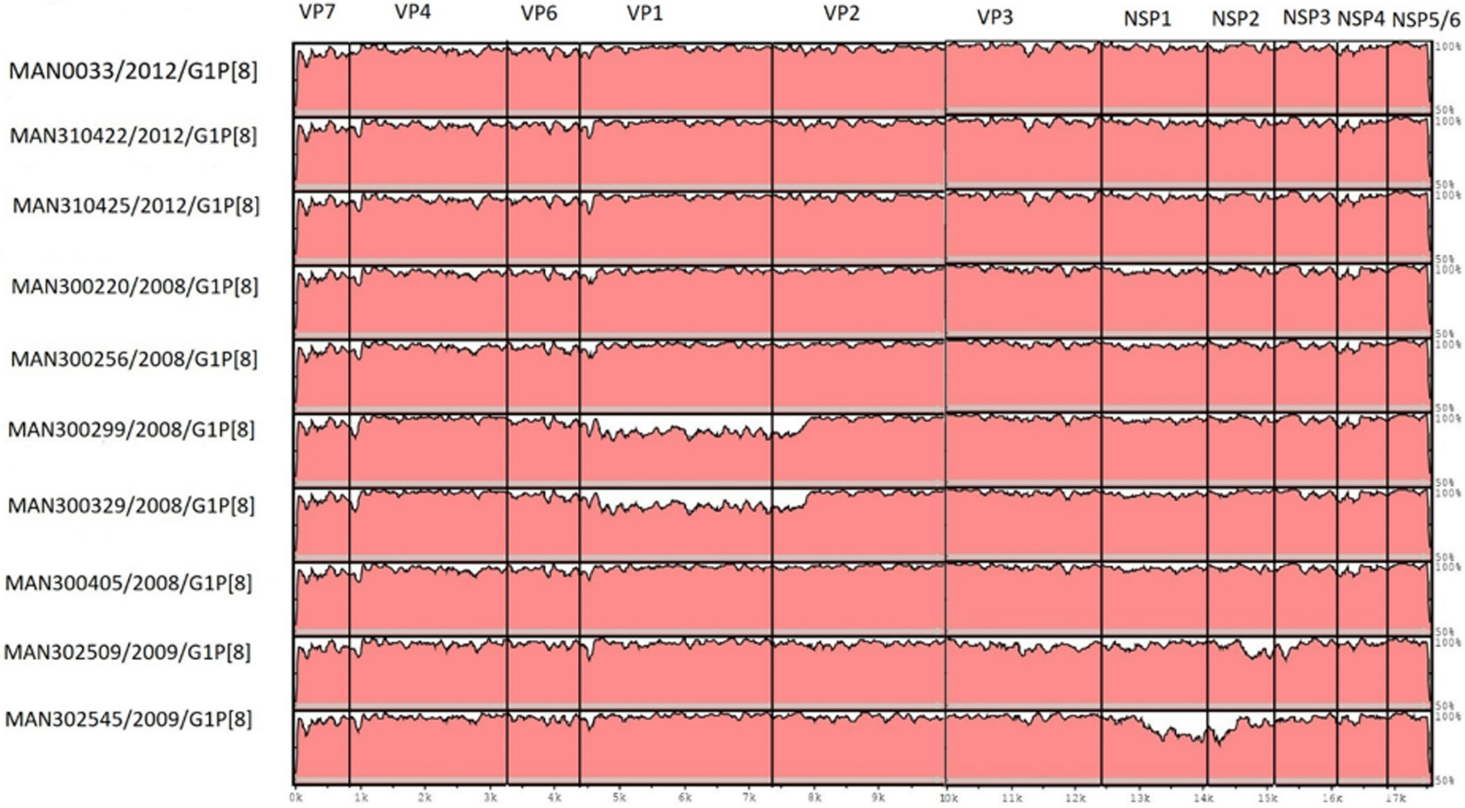
Visualization of the aligned G1P[8] concatenated strains using the mVISTA online server. The post-vaccine strain RVA/Human-wt/MOZ/HCN1552/2017/G1P[8] served as a reference strain. The study strains are described on the left side of the image, and the genome segments are in the top. The image scale indicates the distance in kb.

We observed that in the phylogenetic analysis of the genome segments VP1–VP2, VP6–VP7 and NSP1–NSP5, the G1P[8] study strains clustered separately from Mozambican G1P[8] post-vaccine strains retrieved from GenBank. To unravel the possible role of reassortment events, all G1P[8] concatenated segments (VP7-VP4-VP6-VP1-VP2-VP3-NSP1-NSP2-NSP3-NSP4-NSP5/6) aligned using mVISTA were analysed. In this analysis, the strain MAN0033/2012/G1P[8] was included, as it frequently clustered with the study strains and the post-vaccine strain, MOZ/HCN1552/2017/G1P[8], randomly selected in Excel, was used as a reference strain. The results demonstrated, in general, that all the segments of G1P[8] study strains were not conserved, with variations observed in the VP1 and VP2 genome segments of two strains, one MSD (MAN300329) and one of a child without diarrhoea (MAN300299), NSP2 genome segment of a MSD (302509) and NSP1 and NSP2 genome segments of an MSD (MAN302545) (Fig. 8).

## Discussion

The rotavirus Wa-like genotype constellation is the major group circulating worldwide [16,57]. In this study, we characterized G1P[8] and other Wa-like pre-vaccine strains (G9P[8], G12P[6] and G12P[8]) from children with MSD, LSD and without diarrhoea in Manhiça, Mozambique, with a special focus on the analysis and description of G1P[8] strains. Driven by the fact that the country has adopted the monovalent Rotarix® vaccine composed of the G1P[8] genotype, and which has been reported as the most frequent genotype pre- and post-vaccine introduction in some regions of Mozambique [26,27], it is important to monitor these strains at the genome level.

Phylogenetic analysis of G1 genotypes demonstrated a distinct cluster of the study strains within lineage I, being close to global circulating strains and only with two pre-vaccine strains collected in rural areas in Manhiça (MAN033) in 2012 and in Chokwé (MOZ/21123) in 2011. Analysis of the epitope region of VP7 showed changes in all studied G1 strains and the two Mozambican pre-vaccine strains MAN033 and MOZ/21123 at positions S123N and M217T when compared to the Rotarix® strain. In addition to these two amino acid substitutions, a substitution at position N94S was exclusively observed in all G1 study strains from 2008 to 2009. Changes at these three positions (N94S, S123N and M217T) were previously reported to occur between G1 strains from lineage I, pre-vaccine in Pakistan and India and post-vaccine introduction in Belgium [58,60]. However, few studies have reported the changes we observed at position T91N in one of the study strains [18,61].

The occurrence of mutations at amino acids 94, 147 to 148 and 291, which are involved in antibody recognition and neutralization, is described to impact the host–pathogen interaction [62]. The asparagine residue is N-glycosylated, and a change from asparagine (N) to serine (S), both polar amino acids [63], observed at position 94 could lead to a loss of the glycosylation site, which could impact the immunogenicity of the 7–1a epitope [64]. Likewise, the change at position 217, from methionine (M), which is a non-polar aa, to threonine (T), which is a polar aa, may probably impact the change in the biochemical properties and activity of the protein [63]. The emergence of strains capable of escaping vaccine-induced immunity may be a result of mutations in the VP7 neutralization epitopes [62]. However, this may not be speculated with the study strains, as they are pre-vaccine introduction strains and none of these changes were observed in post-vaccine strains retrieved from the GenBank.

Analysis of P[8] demonstrated distinct clusters of the study strains; although they all fell into lineage III, they were distant from the group of Mozambican strains from 2014 to 2017, from various regions of the country (Nampula, Maputo, Beira and Quelimane), retrieved from GenBank. Even though there is no report of G1 post-vaccine in the study area, there were reports of P[8] combined with other genotypes, such as G3 [30], which were included in the analysis, and phylogenetic analysis demonstrated that they were close to some P[8] strains from 2008. In the analysis of VP4 antigenic regions of the study strains, substitutions were detected at positions E150D, N195G, S125N and N135D, despite these sites being considered conserved [18]. A change from serine (S) to arginine (R) is considered to facilitate the escape of host immunity [63], and this change was observed in all study strains at position 131, as well as in pre- and post-vaccine strains retrieved from the GenBank; however, the role of this change leading to epidemiological fitness of P[8] is still unclear [18]. Nevertheless, the change at position S398E was observed solely in one of the study strains. At position 113, there were changes in the study strains from 2009 and 2012, while post-vaccine P[8] strains from the study area had this position conserved, similar to many strains from pre- and post-vaccine retrieved in the GenBank.

In all the internal genome segments VP1–VP3, VP6 and NSP1–NSP5/6, the study strains had similar evolutionary features regardless of being MSD, LSD or from children without diarrhoea, and they all formed clusters according to the isolation year. Four G1P[8] strains previously circulating in the country in 2012 were found to be more closely related to the study strains, compared to other G1P[8] strains circulating between 2014 and 2017. With the exception of two gene segments, the study strains clustered distant from the post-vaccine strains from the study area.

Alignment using mVISTA suggested that the G1P[8] study strains may have evolved through point mutations. The MAN/302545 strain, which was very distant from the other study strains in the NSP1 genome segment, was confirmed to have likely suffered point mutations in the mVISTA analysis, including in its NSP2 genome segment. The pre-vaccine strain MAN033 (from the same rural area), which was retrieved from the GenBank, previously suggested to be a reassortant strain from the original study [29], was closely related to two G1P[8] MSD study strains (MAN310422 and 310425) that were always in a clade separated from other strains in all of the genome segments, and our results suggest that these strains may have evolved through point mutations.

We observed genetic similarities between strains from children with and without diarrhoea. This suggests that among the study children, host factors, such as poor nutrition [65] and immunosuppression [66], which were not evaluated in this study, may be implicated in the induction of diarrhoeal disease; therefore, these factors are yet to be investigated in future studies. Most critical changes observed in the antigenic region of VP7 were only observed in pre-vaccine strains, in contrast with the changes observed among the antigenic regions of VP4. The data of changes observed in the VP7 epitope in the G1 study strains suggest that a natural fluctuation occurred in these strains. Meanwhile, the mVISTA analysis suggested that the G1P[8] strains in the study area might have suffered point mutations. Although with some limitations, these data suggest a genetic variability of the study strains in this rural location.

One of the study limitations is the lack of continuous data reporting G1P[8] strains in some years (2010–2011) and the absence of G1P[8] strains post-vaccine from the study area, as this would allow comparative analysis, thus providing a broader view of the changes which might have been occurring in G1P[8] strains pre- and post-vaccine introduction. Despite this limitation, the study highlights and provides information on important changes in circulating G1 strains several years pre-vaccine introduction, when compared to G1 strains circulating after Rotarix® vaccine introduction, providing a long-term understanding of the diversity of rotavirus strains in Mozambique. The herein reported changes may represent valuable information for further studies aiming at evaluating the impact of the vaccine on circulating strains and reinforce the need for continued use of WGS as a tool to understand the genetic diversity of rotavirus circulating and emerging genotypes.

## Supplementary material

10.1099/mgen.0.001522 Uncited Supplementary Material 1.

10.1099/mgen.0.001522 Uncited Supplementary Material 2.

## Abbreviations

GEMS: Global Enteric Multicenter Study
LSD: less severe diarrhoea
MSD: moderate-to-severe diarrhoea
RVA: rotavirus group A
VE: vaccine effectiveness
WGS: whole-genome sequencing
