# Supplementary material for: Whole-genome analysis of rotavirus G1P[8] and other Wa-like strains in Mozambican children: evidence of genetic variations of pre-vaccine G1P[8] strains from Manhiça, Mozambique
Source: Microb Genom. 2025 Oct 28;11(10):001522. doi: 10.1099/mgen.0.001522 (PMC12562872; doi:10.1099/mgen.0.001522)
Supplement: Uncited Supplementary Material 1. [file mgen-11-01522-s001.pdf]

Whole-genome analysis of Rotavirus G1P[8] and other Wa-like strains in Mozambican children: Evidence of genetic variations of pre- vaccine G1P[8] strains from Manhiça, Mozambique

Filomena Manjate, Percina Chirinda, Peter Mwangi, Eva D. João, Milton Mogotsi, Marcelino Garrine, Augusto Messa Jr., Delfino Vubil, Nélio Nobela, Karen Kotloff, James P. Nataro, Tacilta Nhampossa, Sozinho Acácio, Goitom Weldegebriel, Jacqueline E. Tate, Umesh Parashar, Jason M. Mwenda, Pedro L. Alonso, Martin Nyaga, Celso Cunha and Inácio Mandomando

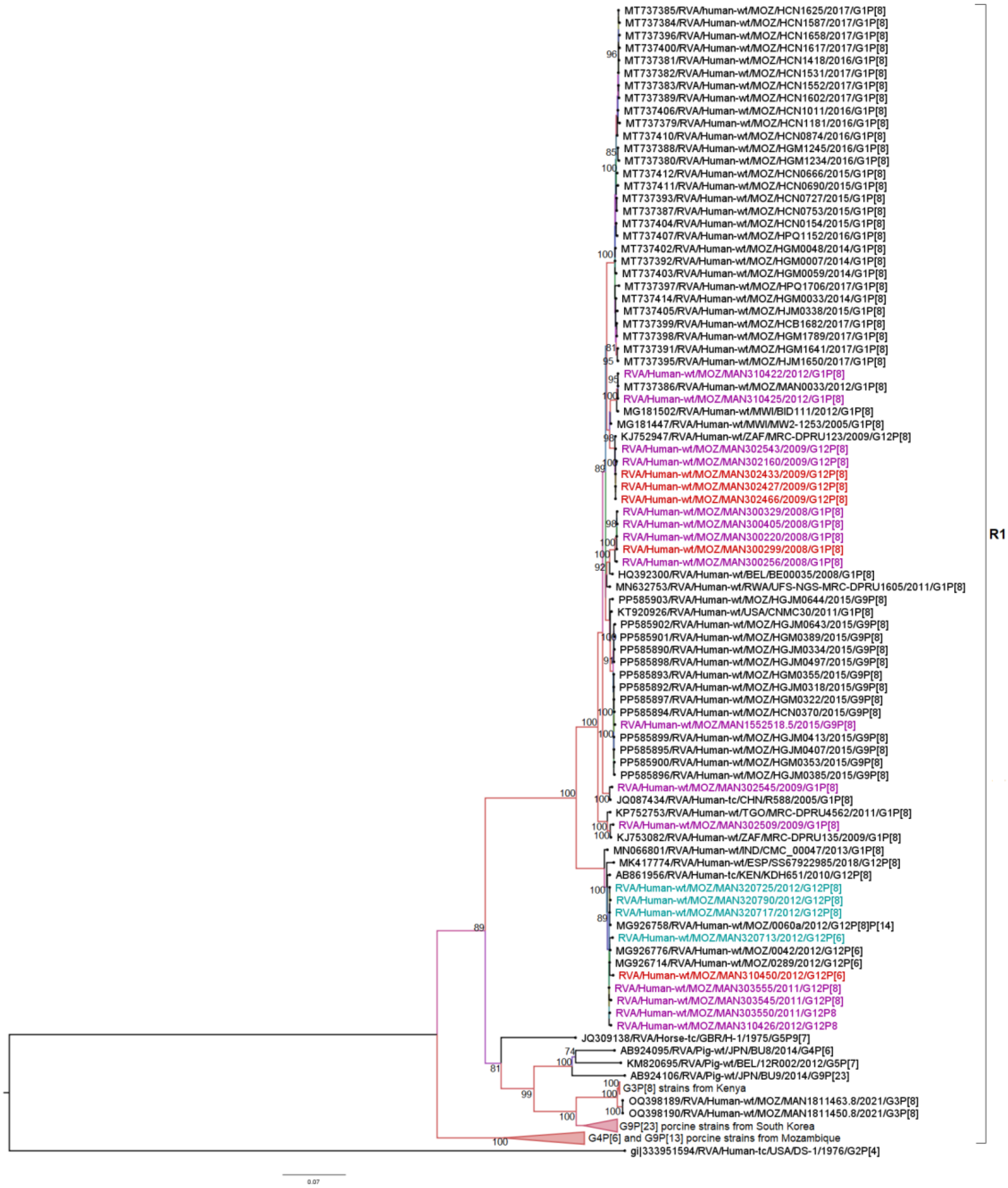

Supplementary Figure 1: **Maximum likelihood phylogenetic tree based on the open reading frames (ORF's) of the VP1 encoding gene segment of the studied strains**. Bootstraps values ≥ 70% are shown adjacent to the nodes. Purple coloured taxa indicate MSD studied strains, green LSD studied strains and red strains of children without diarrhoea. The DS-1 like RVA strain from the USA was included as an out-group.

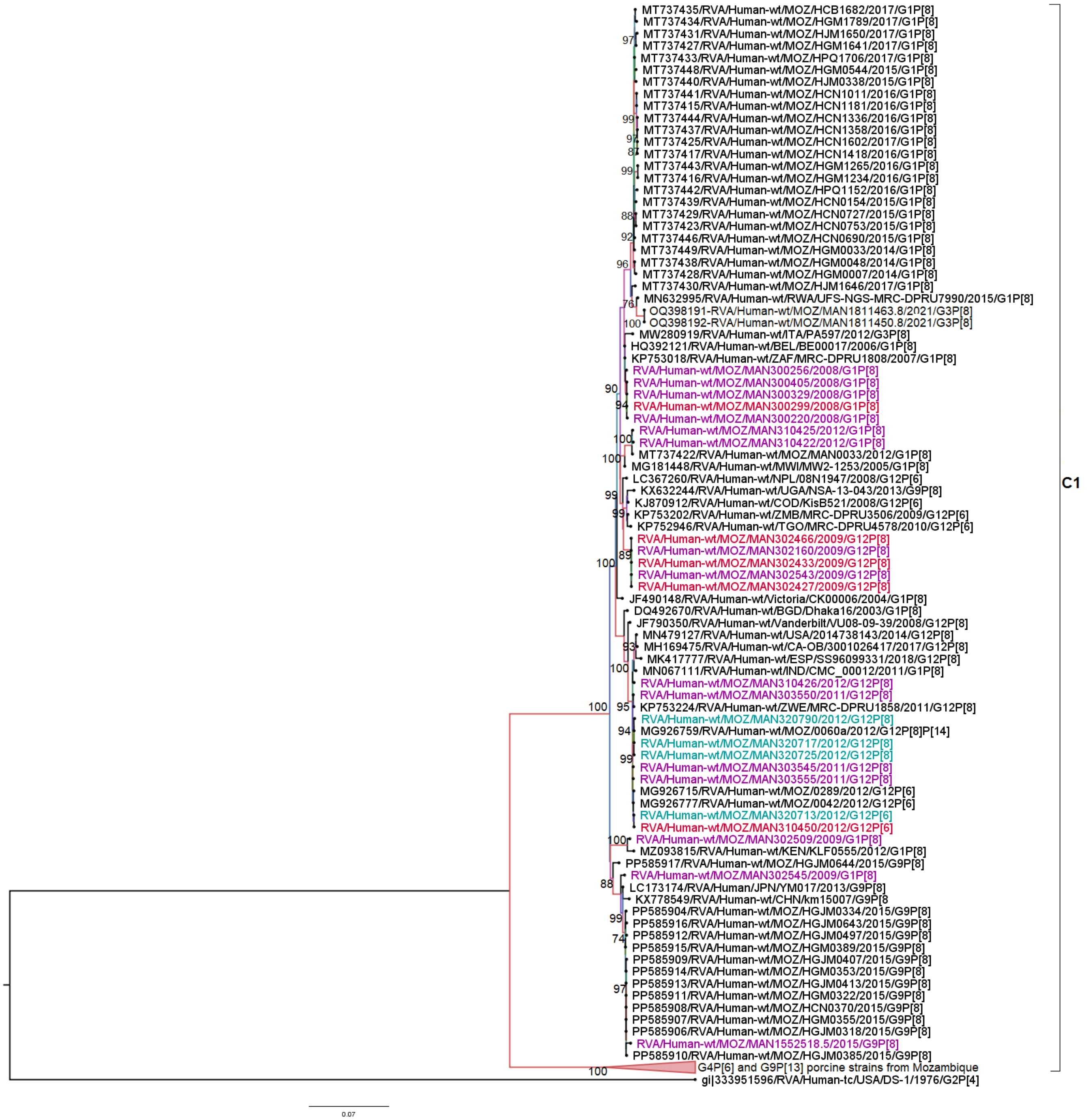

Supplementary Figure 2: **Maximum likelihood phylogenetic tree based on the ORF's of the VP2 encoding gene segment of the studied strains**. Bootstraps values ≥ 70% are shown adjacent to the nodes. Purple coloured taxa indicate MSD studied strains, green LSD studied strains and red strains of children without diarrhoea. The DS-1 like RVA strain from the USA was included as an out-group.

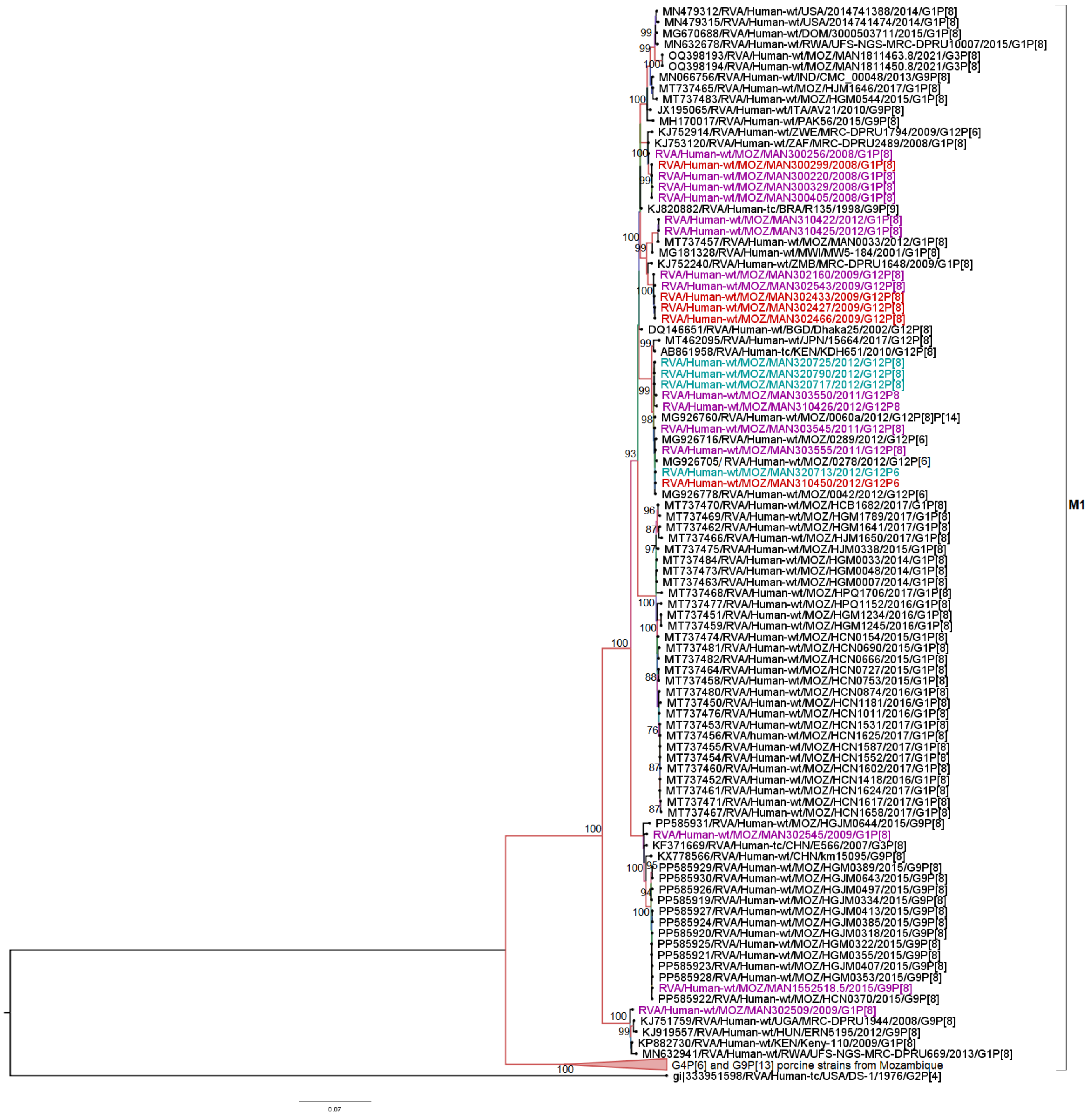

Supplementary Figure 3: **Maximum likelihood phylogenetic tree based on the ORF's of the VP3 encoding gene segment of the studied strains**. Bootstraps values ≥ 70% are shown adjacent to the nodes. Purple coloured taxa indicate MSD studied strains, green LSD studied strains and red strains of children without diarrhoea. The DS-1 like RVA strain from the USA was included as an out-group.

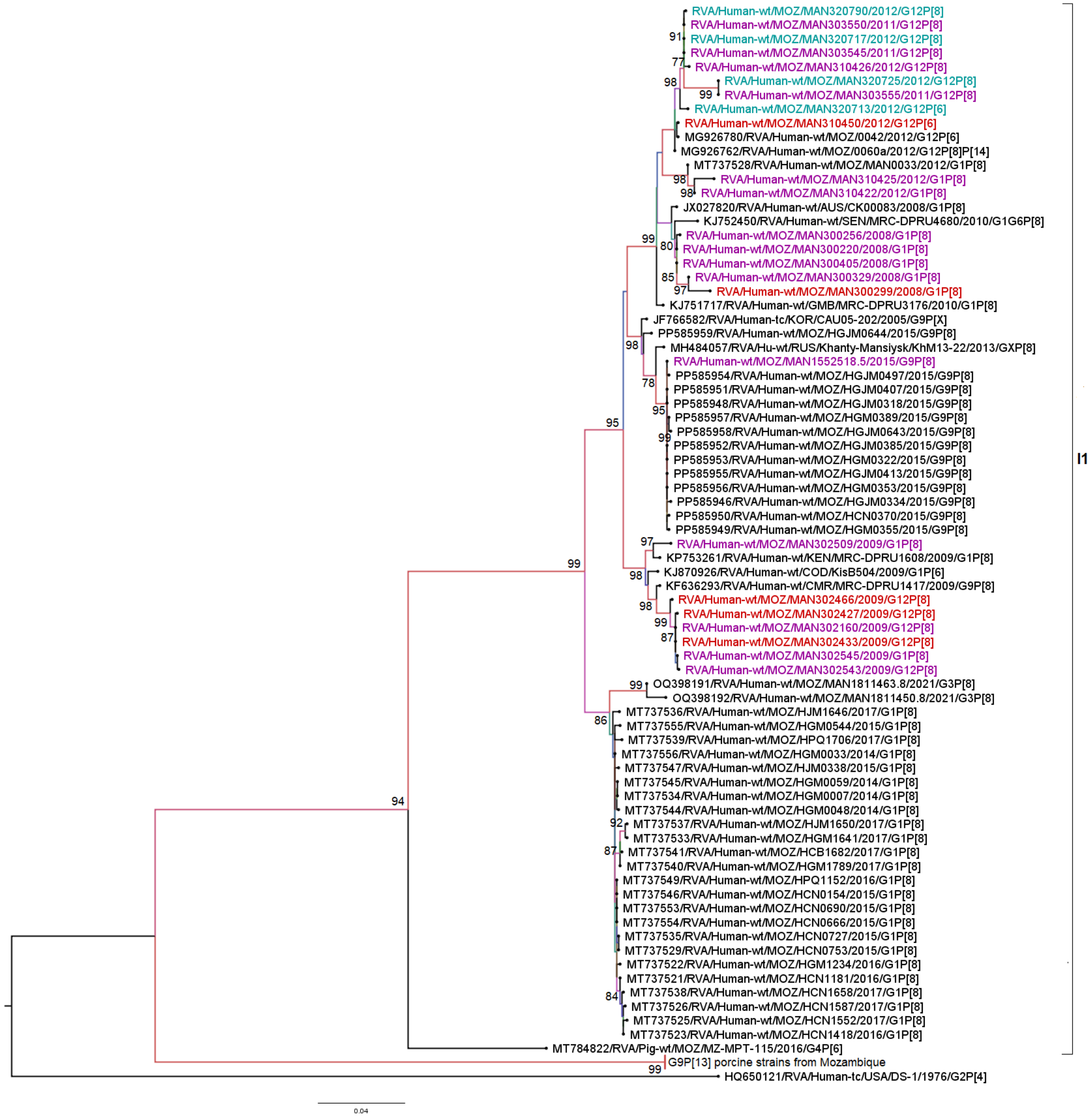

Supplementary Figure 4: **Maximum likelihood phylogenetic tree based on the ORF's of the VP6 encoding gene segment of the studied strains**. Bootstraps values ≥ 70% are shown adjacent to each node. Purple coloured taxa indicate MSD studied strains, green LSD studied strains and red strains of children without diarrhoea. The DS-1 like RVA strain from the USA was included as an out-group.

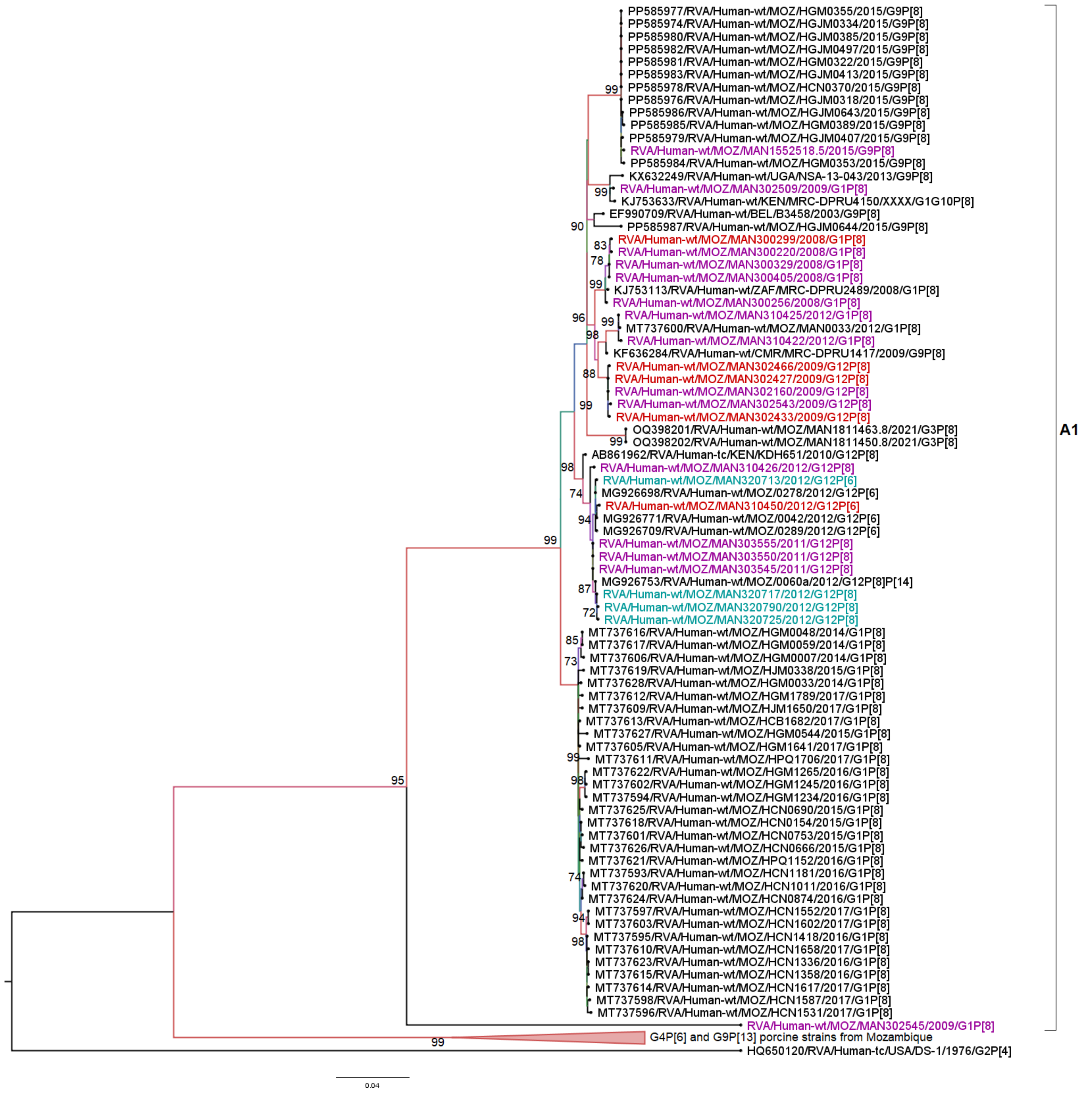

Supplementary Figure 5: **Maximum likelihood phylogenetic tree based on the ORF's of the NSP1 encoding gene segment of the studied strains**. Bootstraps values ≥ 70% are shown adjacent to the nodes. Purple coloured taxa indicate MSD studied strains, green LSD studied strains and red strains of children without diarrhoea. The DS-1 like RVA strain from the USA was included as an out-group.

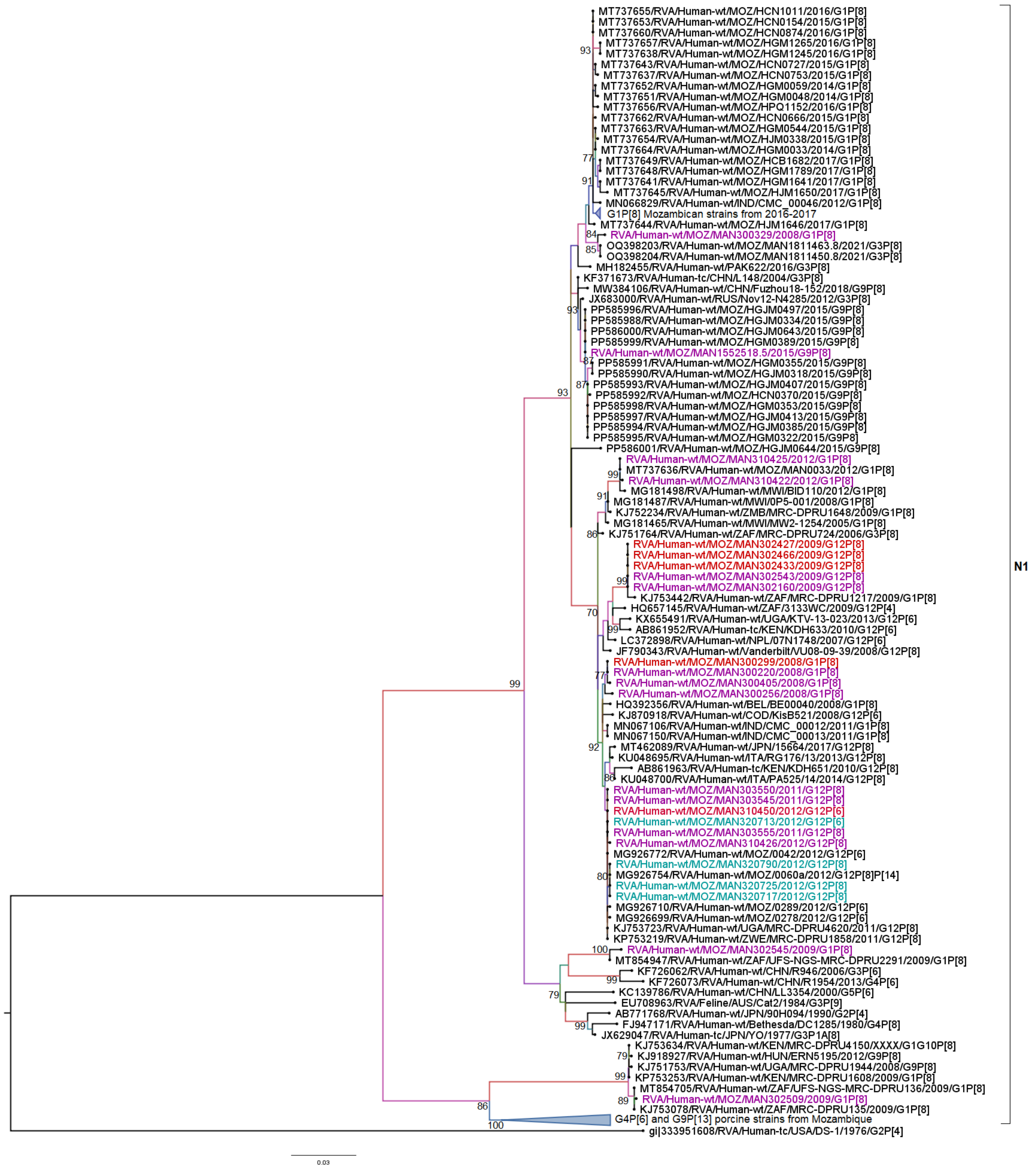

Supplementary Figure 6: **Maximum likelihood phylogenetic tree based on the ORF's of the NSP2 encoding gene segment of the studied strains**. Bootstraps values ≥ 70% are shown adjacent to the nodes. Purple coloured taxa indicate MSD studied strains, green LSD studied strains and red strains of children without diarrhoea. The DS-1 like RVA strain from the USA was included as an out-group.

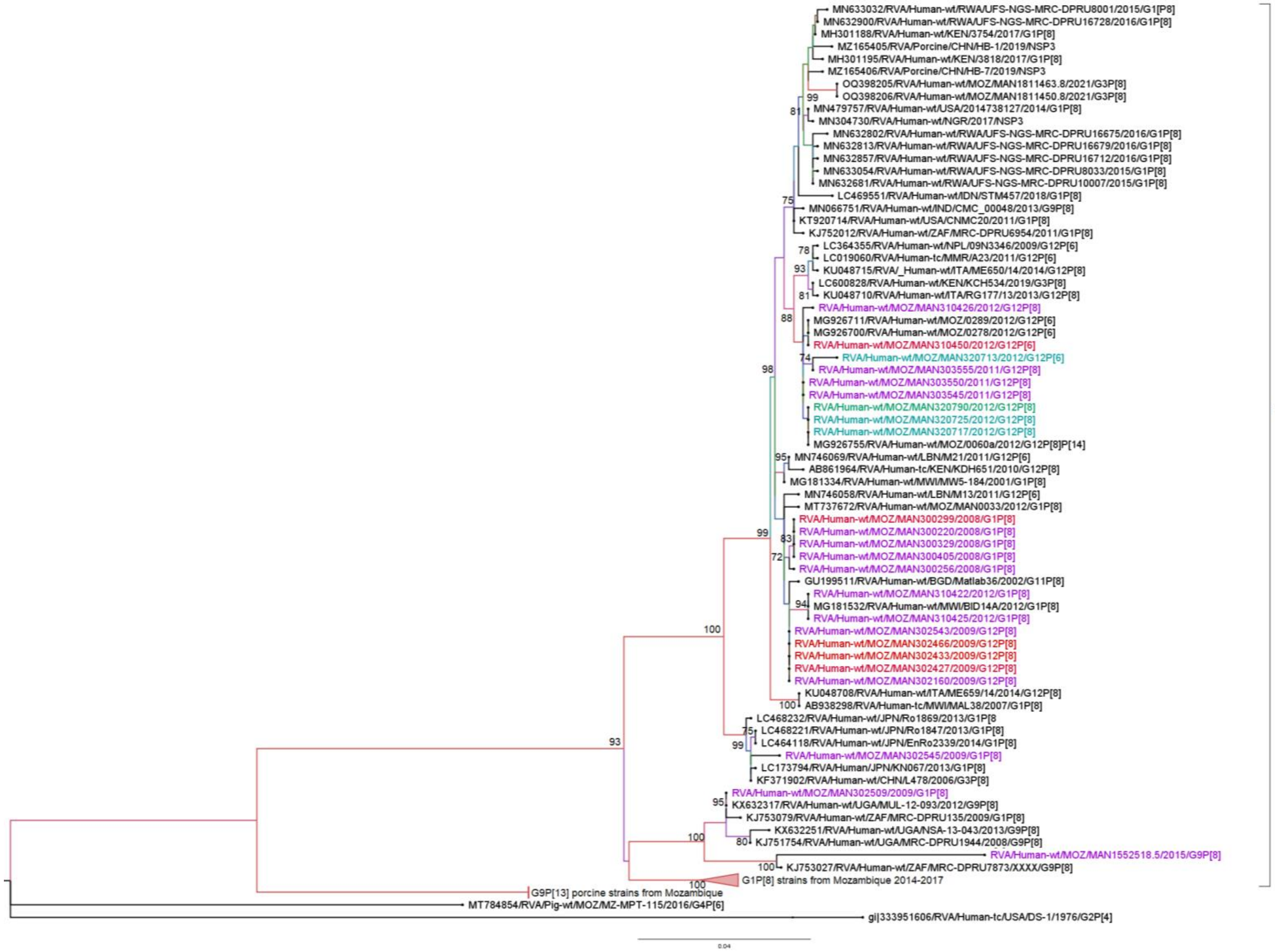

Supplementary Figure 7: **Maximum likelihood phylogenetic tree based on the ORF's of the NSP3 encoding gene segment of the studied strains**. bootstraps values ≥ 70% are shown adjacent to each node. Purple coloured taxa indicate MSD studied strains, green LSD studied strains and red strains of children without diarrhoea. The DS-1 like RVA strain from the USA was included as an out-group.

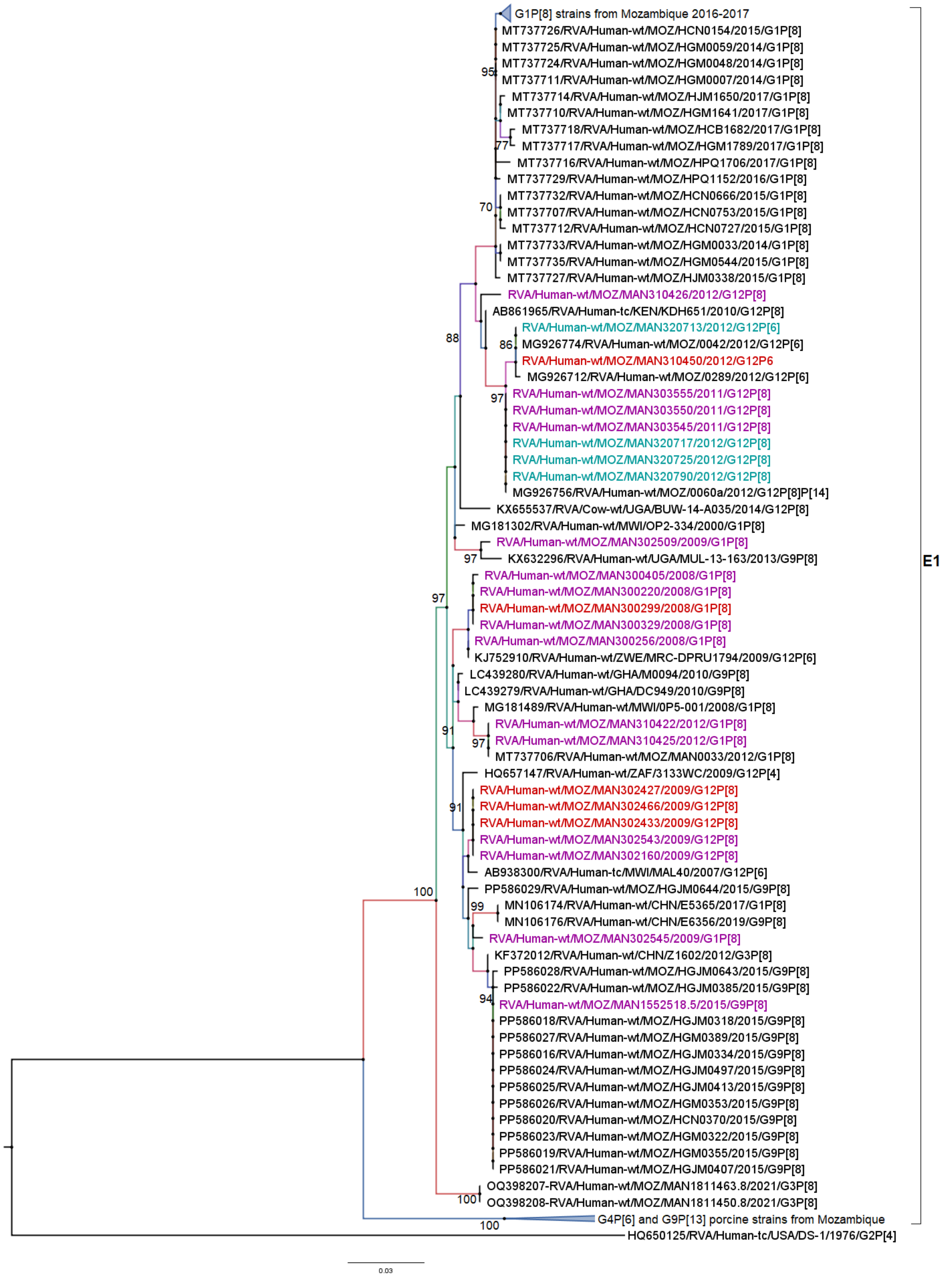

Supplementary Figure 8: **Maximum likelihood phylogenetic tree based on the ORF's of the NSP4 encoding gene segment of the studied strains**. Bootstraps values ≥ 70% are shown adjacent to each node. Purple coloured taxa indicate MSD studied strains, green LSD studied strains and red strains of children without diarrhoea. The DS-1 like RVA strain from the USA was included as an out-group.

.

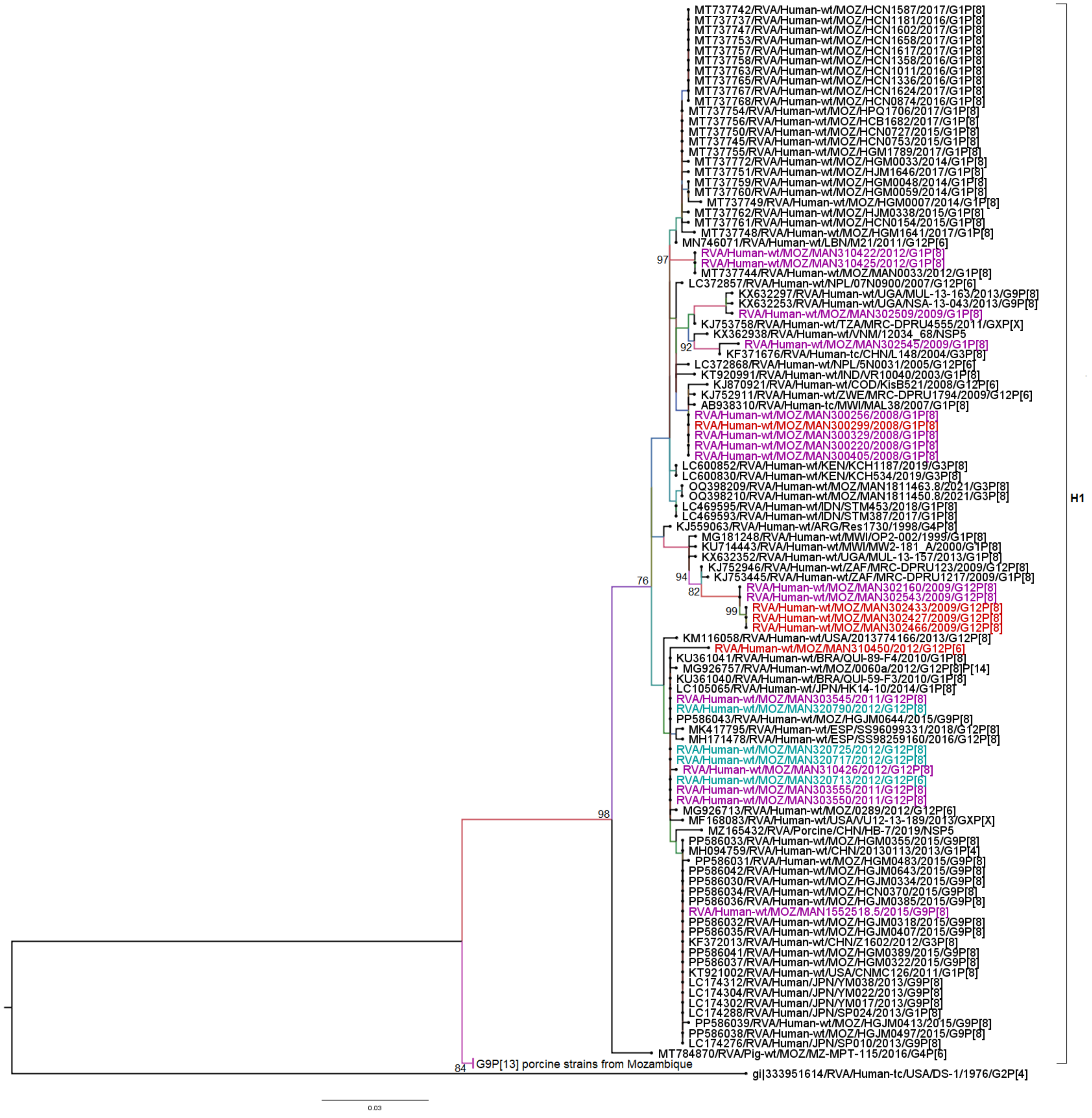

Supplementary Figure 9: **Maximum likelihood phylogenetic tree based on the ORF's of the NSP5/6 encoding gene segment of the studied strains**. Bootstraps values ≥ 70% are shown adjacent to the node. Purple coloured taxa indicate MSD studied strains, green LSD studied strains and red strains of children without diarrhoea. The DS-1 like RVA strain from the USA was included as an out-group.
